# GABPB1-AS1 Promotes the Development of Osteosarcoma by Targeting SP1 and Activating the Wnt/*β*-Catenin Pathway

**DOI:** 10.1155/2022/8468896

**Published:** 2022-03-18

**Authors:** Jingyang Chen, Meiru Bian, Lingxiao Pan, Chengdong Liu, Hanshi Yang

**Affiliations:** ^1^Department of Orthopedics, Ningbo Medical Center Lihuili Hospital, Ningbo 315400, China; ^2^Department of Hematology, The Affiliated Huai'an Hospital of Xuzhou Medical University, Xuzhou, Jiangsu, China; ^3^The Second People's Hospital of Huai'an, Huaian 223002, China; ^4^Department of Orthopedics, Kunshan Geriatric Hospital, Kunshang 215324, China; ^5^Department of Orthopedics, The Affiliated Huai'an Hospital of Xuzhou Medical University, Xuzhou, Jiangsu, China

## Abstract

In this study, the role of GABPB1-AS1 in osteosarcoma (OS) was analyzed. The expression of GABPB1-AS1 in different OS cell lines U2OS, HOS, MG63, and hFOB1.19 was detected. SiRNA GABPB1-AS1 was transfected with U2OS and HOS cell lines. The effects of GABPB1-AS1 silencing on proliferation, clonal formation, and migration of U2OS and HOS were detected by CCK-8 method, plate cloning method, and Transwell chamber. Western blot analysis was used to detect the protein levels of SP1, Wnt, *β*-catenin, c-Myc, and SOX2 in osteosarcoma cells. The binding relationship between GABPB1-AS1 and miR-199a-3p in OS cells was detected by a dual-luciferase reporter gene assay. Results showed that GABPB1-AS1 was higher in OS cells than that in hFOB1.19. Silencing GABPB1-AS1 inhibited the proliferation, clonal formation, migration, and epithelial-mesenchymal transformation of U2OS and HOS. There was a binding relationship between GABPB1-AS1 and miR-199a-3p in OS cells. GABPB1-AS1 mediated osteosarcoma cells via the SP1/Wnt/*β*-catenin signaling pathway. This study suggested that GABPB1-AS1 plays a carcinogenic role in OS through the SP1/Wnt/*β*-catenin signaling pathway through competitive binding and inhibition of miR-199a-3p.

## 1. Introduction

At present, a surgical resection combined with chemotherapy is often used to control tumor metastasis. Therefore, it is very important to seek new therapeutic strategies and therapeutic targets [1].

lncRNA can act as an oncogene or tumor suppressor to regulate the growth and proliferation of cancer cells [2]. lncRNAs and microRNAs (miRNAs) can regulate each other and affect the EMT of tumors [3]. Some lncRNAs play the role of tumor biomarkers in the diagnosis, treatment, and prognosis of osteosarcoma [4].

MicroRNAs are a class of noncoding RNAs (ncRNAs) about 22 nt in length. miRNAs are widely distributed, and their functions almost involve all tumor-related processes, including proliferation, differentiation, apoptosis, metastasis, angiogenesis, and immune response. The mechanism of action of microRNA is relatively simple. It binds to the target gene mRNA3'-untranslated region through complete or partial complementation and regulates the expression of a target gene by degrading or inhibiting mRNA translation. MicroRNA-199a-3p (miR-199a-3p) is one of many microRNAs. Studies [5, 6] have observed its role in liver cancer, ovarian cancer, and osteosarcoma. However, it is rarely studied in osteosarcoma.

SP1 is widely present in tissue nuclei [7]. The SP1 protein functions mainly through the TGF-*β* classical signaling pathway, ERK, PI3K/Akt, and Wnt/*β*-catenin signaling pathway [8, 9]. SP1 can regulate the transcriptional activity and expression of angiogenesis-related factors, oncogenes, and so on [10]. SP1 promotes tumor growth, metastasis, and inhibits apoptosis of tumor cells [11]. Further studies on the role of SP1 and its related signaling pathways in osteosarcoma will be helpful to further understand the mechanism of osteosarcoma occurrence and metastasis [12, 13]. It also provides better and new molecular intervention targets and approaches for the targeted gene therapy of osteosarcoma.

In this study, the expression and effects of GABPB1-AS1 on the clonal formation, migration, and epithelial-mesenchymal transformation of OS cell lines were analyzed. Meanwhile, the binding sites of GABPB1-AS1 and miR-199a-3p and the effects of overexpression of GABPB1-AS1 and upregulation of miR-199a-3p on the Wnt/*β*-catenin signaling pathway were investigated. This study provides reference for the treatment of OS.

## 2. Methods

### 2.1. Cell Culture and Cell Transfection

U2OS, HOS, MG63, and hFOB1.19 cell lines were cultured in a DMEM/F12 medium containing 10% FBS in an incubator at 37°C, 5% CO_2_, and saturated humidity. It was passed once every 2∼3 d. Cells were divided into sh-NC group, sh-GABPB1-AS1 group, NC group (transfected pcDNA-NC), OE group (transfected pcDNA3.1-SP1), NC inhibitor, and miR-199a-3p inhibitor group. When cells grew to 50%∼60%, the knockdown or overexpression plasmid was transfected into U2OS and HOS cells using a Lipofectamine 3000 transfection kit, respectively. All indexes were detected 48 h after transfection.

### 2.2. CCK-8

The cell concentration of each group was adjusted to 3 × 10^4^ cells/mL. A 100 *µ*L cell suspension was inoculated on 96-well plates. 10 *µ*L of CCK-8 solution was added after incubating in an incubator for 72 h. The culture was continued for 2 hours, and the optical density (OD) value at 450 nm was measured on the microplate.

### 2.3. FISH

The cell slipper was placed at the bottom of the 24-well plate, 5 × 10^3^/well. After 24 h culture, the supernatant was removed and the cells were cleaned with 1 × PBS. After fixation with 4% paraformaldehyde, PBS containing 0.5% Triton X-100 was added. The prehybridization solution was closed at 37°C, the lncRNA GABPB1-AS1 probe was hybridized overnight at 37°C, and the cells were cleaned with a hybridization lotion at 42°C under dark conditions. In the hybridization area of DAPI staining sections, the slides were fixed on the slides with sealing tablets under dark conditions. Experimental specimens were observed under laser confocal microscopy.

### 2.4. Clone Formation Experiment

Cells of each group were collected and cultured for 24 h. After trypsin digestion, a suspension with a concentration of 1 × 10^6^ cells/L was prepared. The 6-well plates were inoculated with 1 mL in each well and incubated at 37°C with 5% CO_2_ for 14 d. The culture was terminated when visible clones were formed (the number of cloned cells was about 100). After discarding the medium and washing with PBS two times, 1 mL methanol was added to each well and fixed for 15 min. Then the cells were rinsed slowly with running water, and 1 mL Giemsa dye was added to each well for 30 min. The cells were washed slowly with water and dried in a fume hood. The cells were counted under a microscope and the relative levels of cell cloning were calculated.

### 2.5. Transwell Assay

Cells were taken from each group, and the cell suspension was prepared using a DMEM/F12 medium without FBS. 100 *µ*L cell suspension (1 × 10^8^ cells/mL) was inoculated into the upper compartment. The upper chamber was prepared with Matrigel in advance and cultured at 37°C for 24 h. The lower chamber was fixed. 0.1% crystal violet was dyed for 40 min at room temperature. The excess dye was washed and dried, and the cells passing through the microporous membrane were observed and counted under an optical microscope, and the relative level of cell migration was calculated.

### 2.6. Scratch Test

The cells were cultured to the logarithmic stage and digested with 0.25% trypsin. With the help of a dropper, single cell suspension was blown, cell density was adjusted to 1 × 108/L, and the cell suspension was inoculated on a 6-well plate. Each well was placed at 1 mL under the conditions of 37°C, 5% CO_2_, and saturated humidity. The cells were cultured in the cell incubator for 24 h until the cells were basically fused 6-well plate was scratched vertically using a 100 *μ*L micropipette head. The cells were rinsed twice with PBS solution. The culture plates were placed in an incubator at 37°C with 5% CO_2_ for 12, 24, and 48 h. They were observed by an inverted phase-contrast microscope. In each field, 3 regions were randomly selected and the length of migration scratch space in 100× field was calculated.

### 2.7. Dual-Luciferase Reporter Gene

The GABPB1-AS1 wild-type luciferase reporter gene vector (WT-GABPB1-AS1) containing miR-199a-3p binding site sequence was purchased from Shanghai Jima Pharmaceutical Company. The GABPB1-AS1 mutant luciferase reporter gene vector (MUT-GABPB1-AS1) containing the miR-199a-3p binding site mutation sequence (MUT-GABPB1-AS1) was provided by Shanghai Jima Pharmaceutical Company. Lipofectamine 3000 was used to cotransfect miR-NC and miR-199a-3p mimics with WT-GABPB1-AS1 and MUT-GABPB1-AS1 into U2OS cells. After 48 h, the dual luciferase reporter gene assay system measures the luciferase activity of U2OS cells.

### 2.8. qRT-PCR

A RNA extraction kit was used to extract total RNA from various cells. The reverse transcription kit was used to obtain the cDNA and it was stored in a refrigerator at −20°C until use. Reaction procedure: 94°C 10 min; 94°C 30 s, 62°C 30 s, 62°C 30 s, 40 cycles. The reaction system refers to the qPCR kit instructions. *β*-Actin was used as an internal reference. The 2^−ΔΔCt^ method was used to calculate the relative expression levels of target genes in different cells.

### 2.9. Western Blot

Total protein was extracted from each group by a protein extraction kit. A BCA protein quantification kit was used for protein quantification. Protein were separated on 8% SDS-PAGE gels, transferred onto PVDF membranes (Bio-Rad, Hercules, CA, USA), and blocked for 1 h at room temperature. Monoclonal antibodies of SP1, Wnt, *β*-catenin, c-Myc, SOX2, and GAPDH mice diluted at 1 : 1000 were incubated overnight at 4°C. The film was washed with a TBST buffer and incubated with HRP-labeled goat anti-mouse IgG secondary antibody (1 : 2000) at room temperature for 2 h. The membrane was washed and color was developed with an ECL kit. The comprehensive gel imaging analysis system was utilized to capture pictures. Using GAPDH as an internal reference, the protein expression levels of each group were analyzed.

### 2.10. Statistical Analysis

Statistical analyses were used SPSS 21.0 software. Each group was set up with 3 parallel experiments, which were repeated 3 times. The data were expressed as mean ± standard deviation. The comparison between the two groups was performed by *t*-test. One-way ANOVA was used for comparison between multiple groups, and the SNK-Q test was used for further pairwise comparison. *p* <  0.05 was considered statistically significant.

## 3. Results

### 3.1. Distribution and the Expression Level of GABPB1-AS1

**Figure 1:**
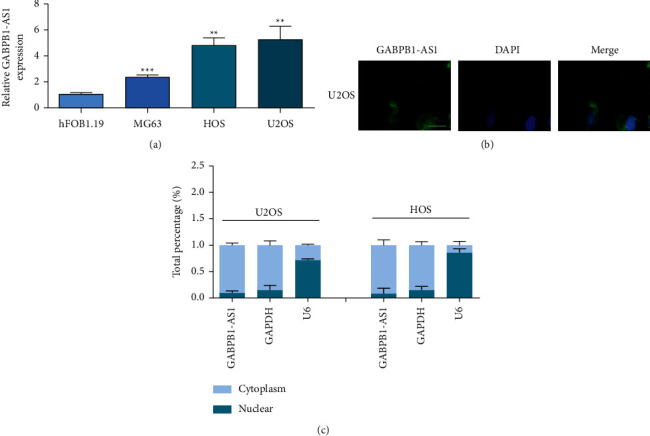
GABPB1-AS1 is upregulated in osteosarcoma cells. (a) qRT-PCR detects the expression profile of GABPB1-AS1 in osteosarcoma cells relative to control FHC cells. (b) FISH shows the subcellular location of GABPB1-AS1 in U2OS cells, scale bar = 10 *μ*m. (c) The subcellular fraction determination shows the subcellular location of GABPB1-AS1 in U2OS and HOS cells. ^*∗∗*^*p* <  0.01 and ^*∗∗∗*^*p* <  0.001.

The relative expression levels of GABPB1-AS1 in hFOB1.19, U2OS, HOS, and MG63 cell lines were analyzed by qPCR. GABPB1-AS1 in each OS cell line was significantly higher than that in normal osteoblast hFOB1.19 cell line (Figure 1(a)). Among them, the relative expression level of GABPB1-AS1 was higher in U2OS and HOS, so U2OS and HOS cell lines were selected for follow-up studies. Fluorescence in situ hybridization and nucleocytoplasmic separation confirmed that GABPB1-AS1 was mainly distributed in the cytoplasm (Figures 1(b)and1(c)).

### 3.2. Effects of GABPB1-AS1 in U2OS and HOS

**Figure 2:**
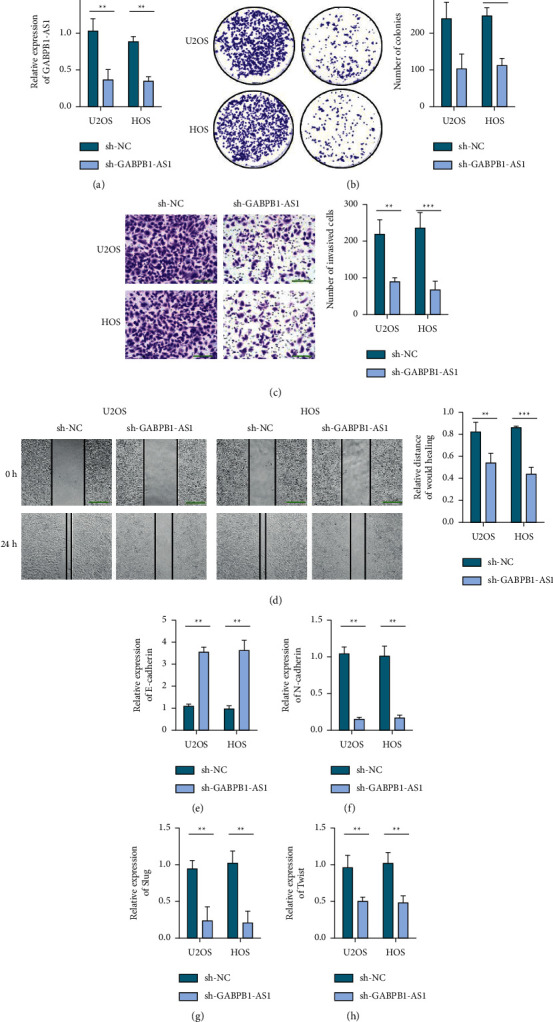
GABPB1-AS1 promotes osteosarcoma cell proliferation, migration, and EMT. (a) Detection of transfection efficiency of GABPB1-AS1 in U2OS and HOS cells. (b) Colony formation experiment to detect the effect of silencing GABPB1-AS1 on the proliferation of osteosarcoma cells. (c) Transwell revealed the invasion ability of GABPB1-AS1-silenced cells, scale bar = 100 *μ*m. (d) Wound healing test shows the migration ability of GABPB1-AS1 silent cells, scale bar = 200 *μ*m. (e) qRT-PCR detects E-cadherin in GABPB1-AS1-silenced cells. (f) qRT-PCR detects N-cadherin in GABPB1-AS1-silenced cells. (g) qRT-PCR to detect Slug in GABPB1-AS1-silenced cells. (h) qRT-PCR detects Twist in GABPB1-AS1-silenced cells.^*∗∗*^*p* <  0.01 and ^*∗∗∗*^*p* <  0.001.

GABPB1-AS1 in the sh-GABPB1-AS1 group was decreased (Figure 2(a)). Compared with the sh-NC group, the survival rate of U2OS and HOS cells, the number of clone formation, and the number of invasive cells were significantly decreased in the sh-GABPB1-AS1 group (Figures 2(b) and 2(c)). Decreased GABPB1-AS1 can inhibit the migration ability of U2OS and HOS cells (Figure 2(d)). After GABPB1-AS1 silencing, E-cadherin was increased, while N-cadherin, Slug, and Twist1 were significantly decreased (Figures 2(e)–2(h)).

### 3.3. SP1 Can Increase the Inhibitory Effect of GABPB1-AS1 in U2OS and HOS

**Figure 3:**
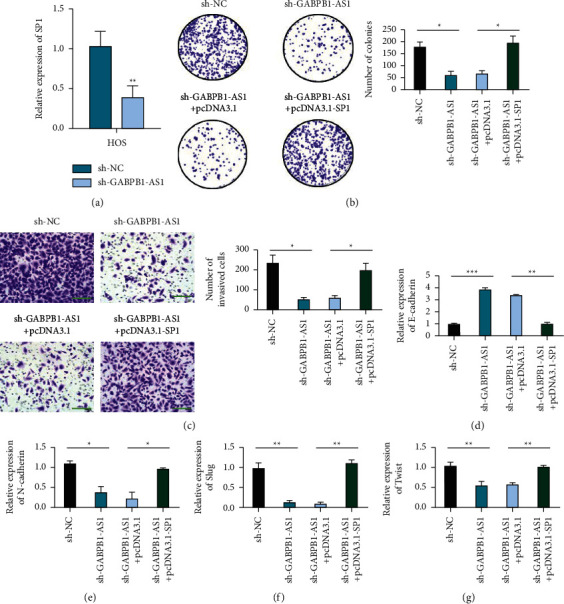
SP1 is essential for the biological function of osteosarcoma cells mediated by GABPB1-AS1. (a) qRT-PCR detects the effect of silencing GABPB1-AS1 on the expression of SP1 in osteosarcoma cells. (b) Colony formation detection of SP1 overexpression on the salvage effect of GABPB1-AS1 deletion on cell proliferation inhibition. (c) Transwell tested the rescue effect of SP1 overexpression on GABPB1-AS1 deficiency that hinders cell invasion, scale bar = 100 *μ*m. (d) qRT-PCR detects the level of EMT biomarker E-cadherin in different treated cells. (e) qRT-PCR detects the level of EMT biomarker N-cadherin in different treated cells. (f) qRT-PCR detects the level of EMT biomarker Slug in different treatment cells. (g) qRT-PCR detects the level of EMT biomarker Twist in different treatment cells.^*∗*^*p* <  0.05, ^*∗∗*^*p* <  0.01, and ^*∗∗∗*^*p* <  0.001.

SP1 in the sh-GABPB1-AS1 group was decreased (Figure 3(a)). In U2OS and HOS cells, the sh-GABPB1-AS1 + pcDNA3.1-SP1 group showed an enhanced clone-forming ability and invasion ability compared with the sh-GABPB1-AS1 + pcDNA3.1-NC group (Figures 3(b) and 3(c)). In U2OS and HOS cells, the expression of E-cadherin in the sh-GABPB1-AS1 + pcDNA3.1-SP1 group was decreased compared with that in the sh-GABPB1-AS1 + pcDNA3.1-NC group while N-cadherin, Slug, and Twist1 expression levels increased (Figures 3(d)–3(g)).

### 3.4. GABPB1-AS1 Targets miR-199a-3p

**Figure 4:**
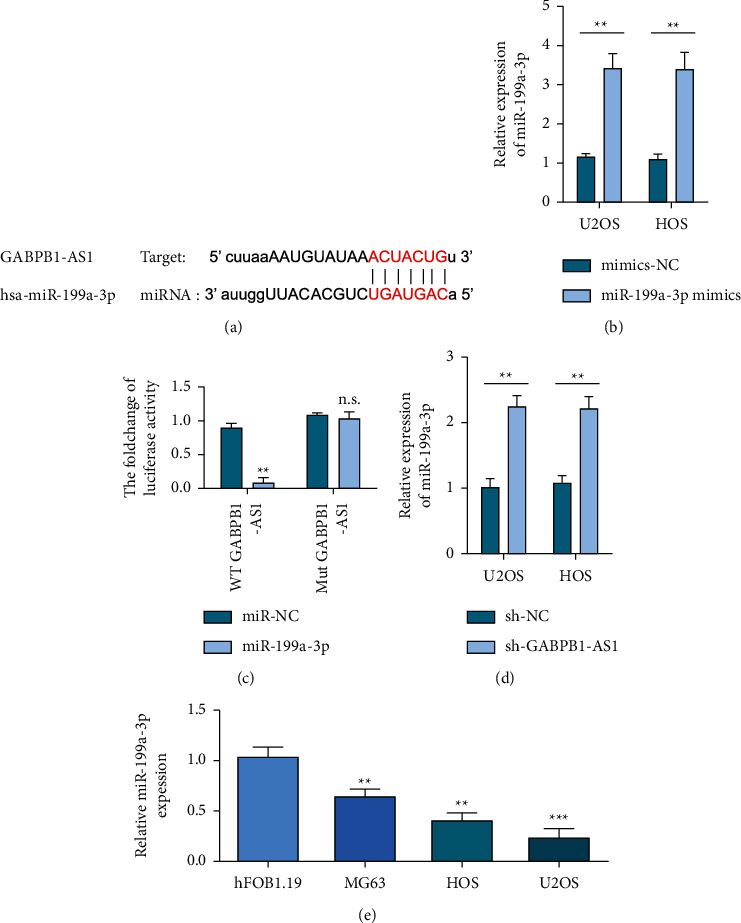
GABPB1-AS1 binds to miR-199a-3p. (a). StarBase predicts the binding site of GABPB1-AS1 and miR-199a-3p. (b) The transfection efficiency of hsa-miR-199a-3p mimics. (c) Using miR-199a-3p mimics to detect the luciferase activity of wild-type and mutant GABPB1-AS1 in osteosarcoma cells. (d) qRT-PCR revealed the effect of GABPB1-AS1 on the expression of miR-199a-3p. (e) qRT-PCR analysis of the expression profile of miR-199a-3p in osteosarcoma cells and control cells.^*∗∗*^*p* <  0.01 and ^*∗∗∗*^*p* <  0.001.

Bioinformatics analysis showed that there were partial continuous complementary nucleotide sequences between GABPB1-AS1 and miR-199a-3p (Figure 4(a)). The dual-luciferase report assay showed that the luciferase activity in the miR-199a-3p mimics and WT-GABPB1-AS1 cotransfection group was significantly lower than that in the miR-NC and WT-GABPB1-AS1 cotransfection group. However, the luciferase activity of U2OS and HOS cells in the miR-199a-3p mimics and MUT-GABPB1-AS1 cotransfection group showed no change compared with that in the miR-NC and MUT-GABPB1-AS1 cotransfection group (Figures 4(b)and 4(c)). miR-199a-3p in U2OS and HOS cells in the sh-GABPB1-AS1 group was increased (Figure 4(d)). miR-199a-3p in OS cell lines was lower than that in normal osteoblast hFOB1.19 cell line (Figure 4(e)).

### 3.5. Inhibition of miR-199a-3p Can Reverse the Effects of GABPB1-AS1 on Proliferation, Migration, and Invasion of U2OS and HOS

**Figure 5:**
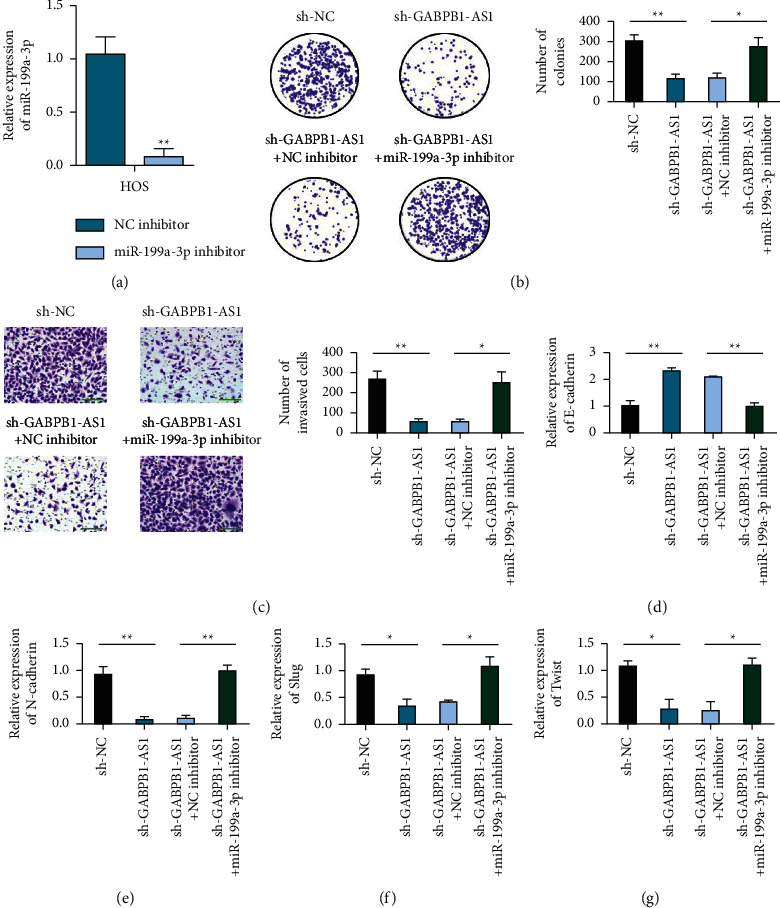
Inhibition of miR-199a-3p reversed the carcinogenic effect of GABPB1-AS1. (a) Detection of miR-199a-3p transfection efficiency. (b) Colony formation tested the rescue effect of inhibiting miR-199a-3p on GABPB1-AS1 deficiency and inhibiting cell proliferation. (c) Transwell tested the salvage effect of inhibiting miR-199a-3p on GABPB1-AS1 deficiency and preventing cell invasion, scale bar = 100 *μ*m. (d) qRT-PCR detects the level of EMT biomarker E-cadherin in different treated cells. (e) qRT-PCR detects the level of EMT biomarker N-cadherin in different treated cells. (f) qRT-PCR detects the level of EMT biomarker Slug in different treatment cells. (g) qRT-PCR detects the level of EMT biomarker Twist in different treatment cells.^*∗*^*p* <  0.05 and ^*∗∗*^*p* <  0.01.

Expression of miR-199a-3p in the miR-199a-3p inhibitor group was decreased (Figure 5(a)). In U2OS and HOS cells, the clonogenic and invasive abilities of the sh-GABPB1-AS1 + miR-199a-3p inhibitor group were enhanced compared with those of the sh-GABPB1-AS1 + NC inhibitor group (Figures 5(b) and 5(c)). In U2OS and HOS cells, E-cadherin expression was decreased in the sh-GABPB1-AS1 + miR-199a-3p inhibitor group compared with the sh-GABPB1-AS1 + NC inhibitor group. In contrast, N-cadherin, Slug, and Twist1 expression levels increased (Figures 5(d)–5(g)).

### 3.6. Osteosarcoma Cells Were Mediated by GABPB1-AS1 via the SP1/Wnt/*β*-Catenin Signaling Pathway

**Figure 6:**
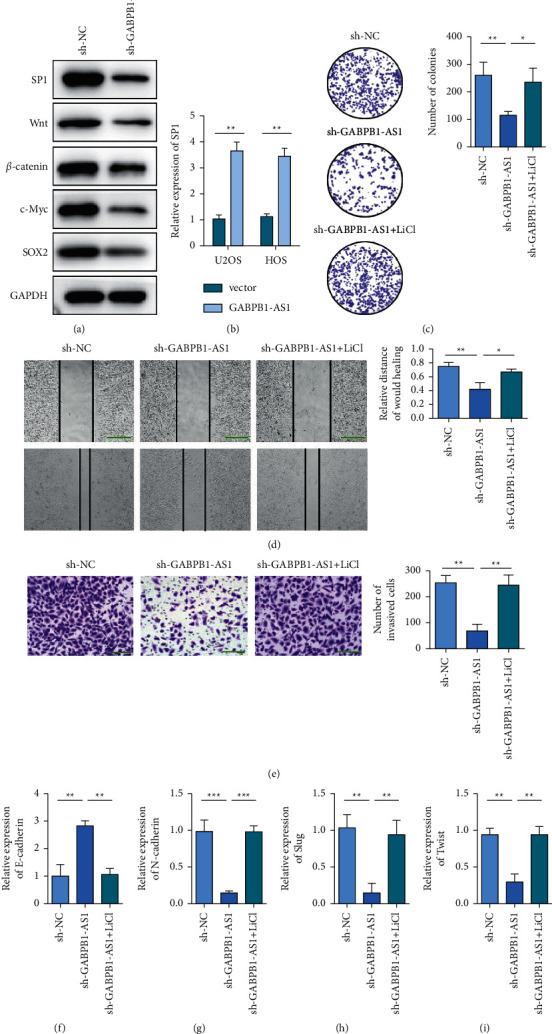
GABPB1-AS1 mediated osteosarcoma cells through the SP1/Wnt/*β*-catenin signaling pathway. (a) Western blot to detect the protein levels of SP1, Wnt, *β*-catenin, c-Myc, and SOX2 in osteosarcoma cells when GABPB1-AS1 is silent or not. (b) Colony formation to evaluate the rescue effect of LiCl treatment on the proliferation of GABPB1-AS1-silenced osteosarcoma cells. (c) Overexpression of lncRNA GABPB1-AS1 can upregulate the expression of SP1. (d) Wound healing test to evaluate the rescue effect of LiCl treatment on the migration of GABPB1-AS1 silenced osteosarcoma cells, scale bar = 200 *μ*m. (e) Transwell evaluates the rescue effect of LiCl treatment on the invasion of GABPB1-AS1 silenced osteosarcoma cells, scale bar = 100 *μ*m. (f) qRT-PCR detects the level of EMT biomarker E-cadherin in different treated cells. (g) qRT-PCR detects the level of EMT biomarker N-cadherin in different treatment cells. (h) qRT-PCR detects the level of EMT biomarker Slug in different treatment cells. (i) qRT-PCR detects the level of EMT biomarker Twist in different treated cells. ^*∗*^*p* <  0.05, ^*∗∗*^*p* <  0.01, and ^*∗∗∗*^*p* <  0.001.

Western blot was used to detect the protein levels of SP1, Wnt, *β*-catenin, c-Myc, and SOX2 in osteosarcoma cells with or without GABPB1-AS1 silencing. The results showed that silencing GABPB1-AS1 decreased the protein levels of SP1, Wnt, *β*-catenin, c-Myc, and SOX2 (Figure 6(a)). The experimental results of Figure 6(b) showed that overexpression of GABPB1-AS1 could upregulate SP1 expression. Clonal formation to evaluate the salvage effect of LiCl treatment on proliferation of GABPB1-AS1-silenced osteosarcoma cells [14]. Compared with the sh-GABPB1-AS1 group, the clone formation ability of the sh-GABPB1-AS1 + LiCl group was enhanced (Figure 6(c)). The wound healing assay and Transwell assay were used to evaluate the effects of LiCl treatment on the migration and invasion of GABPB1-AS1-silenced osteosarcoma cells. Compared with the sh-GABPB1-AS1 group, sh-GABPB1-AS1 + LiCl group showed enhanced cell migration and invasion ability (Figures 6(d)and6(e)). In U2OS and HOS cells, the expression of E-cadherin was decreased in the sh-GABPB1-AS1 + LiCl group compared with the sh-GABPB1-AS1 group. In contrast, N-cadherin, Slug, and Twist1 expression levels increased (Figures 6(f)–6(i)). These results indicate that GABPB1-AS1 mediated the EMT process of osteosarcoma cells through SP1/Wnt/*β*-catenin signaling pathway.

## 4. Discussion

The proliferation and local invasion of osteosarcoma cells are the key stages of tumorigenesis and development [15]. Exploring the molecular mechanism of proliferation and migration of osteosarcoma for identifying new therapeutic targets and developing new therapeutic strategies was important [16, 17].

There are a large number of lncRNAs with an abnormal expression in osteosarcoma tissues, which play an important role in transcription and posttranscription levels [18]. lncRNAs regulate the proliferation, apoptosis, invasion, migration, drug resistance, and other malignant biological behaviors of osteosarcoma [19, 20]. Zhu et al. [21] analyzed 5 pairs of osteosarcoma and paracancer tissues by lncRNA microarray and screened differentially expressed lncRNAs. Results showed that 65 lncRNAs were upregulated and 13 lncRNAs were downregulated. It was further found that lncRNA nucleolar small RNA host gene 16 (ENSG00000163597) had the highest expression level in osteosarcoma. Studies showed that host gene 16 of small nucleolar RNA (ENSG00000163597) can act as an endogenous sponge for miR-205, upregulating the expression of zinc finger e-box zinc finger protein 1 and significantly enhancing the proliferation ability of osteosarcoma cells. Li et al. [22] found that lncRNA actin fibro-associated protein 1 (AFAP1)-AS1 expression was significantly upregulated in osteosarcoma tissues and cell lines. AFAP1-AS1 can capture miR-4695-5p through sponging, thus upregulating the expression of transcription factor 4. Transcription factor 4, as a key transcription factor in the Wnt/*β*-catenin pathway, promotes the proliferation of osteosarcoma.

GABPB1-AS1 in osteosarcoma tissues was significantly higher than that in adjacent tissues. The high expression of GABPB1-AS1 may be related to the progression of osteosarcoma. A functional loss assay showed that the survival rate, clonal formation, and migration of U2OS and HOS cells were decreased after the GABPB1-AS1 expression was inhibited by transfection with GABPB1-AS1 small interfering RNA. E-cadherin can maintain epithelial cell morphology and tissue integrity. Conversely, it promotes metastasis and diffusion of tumor cells. Studies have shown that lncRNA TTTYl5 knockdown can reduce the invasion ability of osteosarcoma cells. N-cadherin expression was decreased and E-cadherin was significantly increased in U2OS and HOS cells after GABPB1-AS1 expression was inhibited, which was consistent with the results of functional loss experiment. These results suggest that GABPB1-AS1 plays an oncogene role in osteosarcoma. In recent years, a number of studies have shown that lncRNAs can play a role in tumors by targeting miRNA and blocking its function. For example, FGD5-AS1 regulates the growth, migration, invasion, and apoptosis of esophageal squamous cell carcinoma by targeting miR-383 [23]. FGD5-AS1 increases the ability of malignant proliferation, migration, and invasion of colorectal cancer cells by interacting with miR-302e [24]. This study found that miR-199a-3p may interact with GABPB1-AS1. miR-199a-3p is a member of the miR-family, and previous studies have shown that miR-199a-3p has a low expression in OS [25]. miR-199a can inhibit the of non-small-cell lung cancer by regulating HIF-1*α* [26]. miR-199a-3p can induce metastasis of malignant tumor cells by inhibiting the expression of SMARCA2, downregulating the expression of SMARCA4, and inhibiting the expression of TGF-*β*. TGF-*β* also regulates epithelial-mesenchymal transformation and participates in tumor cell metastasis [27].

In this study, dual-luciferase assay confirmed that GABPB1-AS1 had a negative regulatory effect on miR-199a-3p. In addition, the inhibition of GABPB1-AS1 expression could increase miR-199a-3p. Further functional analysis showed that overexpression of miR-199a-3p enhanced inhibition of GABPB1-AS1 on proliferation, migration, apoptosis, and related protein expression of U2OS and HOS cells. Inhibition of miR-199a-3p expression reversed the effects of GABPB1-AS1 inhibition on proliferation, migration, apoptosis, and related protein expression of U2OS and HOS cells. The abovementioned studies indicate that the GABPB1-AS1/miR-199a-3p pathway in osteosarcoma and the exploration of downstream target genes and possible signaling pathways of miR-199a-3p is the next research focus.

The occurrence and metastasis of tumors are highly correlated with abnormal activation of the Wnt signaling pathway [28]. In particular, lncRNA dysregulation can activate Wnt signal transduction pathways, leading to cancer growth and metastasis. Eight members of the SP family (SP1∼SP8) are a highly evolutionarily related transcription factor [29]. SP1 was first discovered in this family, and its gene, located at 12q1, is widely expressed in various tissues. SP1 affects the malignant tumors from different tissues [30]. In this study, we found that GABPB1-AS1 promotes the development of osteosarcoma by targeting SP1 to activate the Wnt/*β*-catenin pathway.

## 5. Conclusion

In summary, GABPB1-AS1 was highly expressed in osteosarcoma. The inhibition of GABPB1-AS1 can reduce the proliferation and migration of osteosarcoma cells. Mechanism studies showed that lncRNA GABPB1-AS1, as a competitive endogenous RNA, upregulated the expression of SP1 and promoted the Wnt/*β*-catenin pathway through sponge adsorption of miR-199a-3p, ultimately promoting the proliferation and malignant evolution of osteosarcoma.

## Data Availability

The analyzed datasets generated during the study are available from the corresponding author on reasonable request.
